# Subterranean Acoustic Activity Patterns of *Vitacea polistiformis* (Lepidoptera: Sesiidae) in Relation to Abiotic and Biotic Factors

**DOI:** 10.3390/insects10090267

**Published:** 2019-08-23

**Authors:** Edidiong I. Inyang, Raymond L. Hix, Violeta Tsolova, Barukh B. Rohde, Omotola Dosunmu, Richard W. Mankin

**Affiliations:** 1 Center for Biological Control, Florida A&M University (FAMU), Tallahassee, FL 32307, USA; 2 Center for Viticulture, FAMU, Tallahassee, FL 32307, USA; 3 Electrical and Computer Engineering Department, University of Florida, Gainesville, FL 32611, USA; 4 Entomology and Nematology Department, University of Florida, Gainesville, FL 32611, USA; 5 US Department of Agriculture, Agricultural Research Service, Center for Medical, Agricultural and Veterinary Entomology, Gainesville, FL 32608, USA

**Keywords:** grape root borer, vineyard, Florida

## Abstract

Grape root borer (GRB), *Vitacea polistiformis*, is a root-feeding pest of grapevines in the US southeast that causes underground damage well before vines show visible symptoms. A 269-d study was conducted at 31 sites in a Florida vineyard to record short bursts of insect movement and feeding vibrations in grapevine root systems and provide information that can improve timing and targeting of GRB management efforts. Characteristic spectral and temporal patterns in the subterranean vibrations facilitated discrimination of GRB from background noise and non-targeted arthropods. Infestation likelihood of GRB at each site was estimated from previous studies relating infestation to burst rate. In all, 39% of recordings indicated low infestation likelihood. Sites with medium or high infestation likelihood were confined to a small region of the vineyard where a vine with larval feeding damage was confirmed. The restricted area suggests that the biological control or chemical treatments could be reduced elsewhere. Acoustic activity was significantly greater in fall and winter than in spring, and greater in evening than afternoon; fall evenings seemed best for GRB acoustic surveys. The GRB seasonal and circadian acoustic variation reflected phenological variation in grape root growth and nutrients and was not significantly correlated with temperature.

## 1. Introduction

*Vitacea polistiformis* Harris (Lepidoptera: Sesiidae) larvae feed on grapevine roots in much of the southeastern US [1,2,3,4], reducing vineyard yields and increasing the susceptibility of vines to pathogens and drought [5]. Due to the high cost of vine replacement, Dutcher and All [6] estimated the economic injury threshold to be 0.074 larvae/vine, with one larva feeding where the roots meet the trunk causing as much as a 47% decrease in fruit yield from the vine the following year.

Several strategies have been investigated for *V. polistiformis* management, including chemical control with Lorsban and mating disruption [3,7,8,9]. Cultural practices that have been used for control of *V. polistiformis* include weed management to reduce survival of neonates attempting to burrow underground after dropping from eggs laid on or near grape foliage, mounding of soil beneath the vines after larvae have pupated, and covering of soil under vines with ground cloth to impede movement of newly emerged adults from subterranean cocoons to the ground surface [7].

None of these strategies are considered to be highly effective against *V. polistiformis* in Florida [2,4,10], partly due to difficulty in timing the peaks of neonate emergence as well as the period when larvae tunnel upwards from the root system to pupate near the surface. Lorsban can be applied once per season, for example, but, because the period of adult *V. polistiformis* flight and oviposition lasts 3–5.5 months in Florida, Lorsban can control only the portion of the neonate population within a few centimeters of the ground surface during its short, 3–4-weeks period of effectiveness [10]. Attracted to grape root volatiles, the neonates move belowground soon after emerging [11]. In addition, maturing larvae may tunnel up into the mounded ridge before pupating if mounding has been done too early in the season [12].

Biological control methods, including predation by numerous bird species [13], are potential adjuncts to chemical and cultural control strategies. Dutcher and All [14] reported that firefly larvae, *Photuris pennsylvanica* De Geer, and soldier beetle larvae, *Chauliognathus pennsylvanicus* De Geer, feed on *V. polistiformis* as well as tiger beetles, *Cicindella punctulata* (Olivier); ground beetles, *Calosoma sayi* (DeJean), *Harpalus pennsylvanicus* (De Geer), *Calathus* sp., shore flies *Notiophilus* spp., and staphylinids. The parasitoid, *Bracon caulicola* (Gaham), has been reported to attack mature larvae within the top 5 cm of soil [15]. Pupae were attacked by *C. pennsylvanicus* and fungi, *Aspergillus flavus* Link ex Fr. [14], as well as *Beauveria bassiana* Balsamo, and green muscardine fungus, *Metarrhizium anisopliae* Metchnikoff [13,16].

Entomopathogenic nematodes also have been of interest for *V. polistiformis* larval control. All et al. [17] reported the initial use of *Steinernema carpocapse* (Weiser) in several Concord grape vineyards in Georgia. They reported low levels of larval and pupal mortality, however, and suggested that naturally occurring nematode populations would not be large enough for proper control; consequently, the natural populations would need to be augmented. In later studies, application of predatory nematodes of the genera *Heterorhabditis* and *Steinernema* showed promising results against *V. polistiformis* larvae [18,19]. Hix [20,21] reported that *Heterorhabditis bacteriophora* (Poinar) and *H. megidis* Poinar, Jackson and Klein can control *V. polistiformis*. Said [19] found that both species were effective when delivered through drip irrigation.

Examining combinations of chemical and biological control methods, Dutcher and All [14] considered the effects of carbaryl (Sevin) and methyl parathion on naturally occurring biological control agents in vineyards. Egg predation by natural predators and egg hatchability were 11.6% and 25.3%, respectively, in a treated vineyard compared to 61.7% and 76.38%, respectively, in an untreated vineyard. The life stages with the greatest percentages of mortality were reported to be the eggs and neonates [14].

The effectiveness of the above chemical, cultural, physical, and biological control methods would benefit from improved understanding of larval spatial distributions within fields and more precise knowledge of the timing of larval and pupal development [2,3,4,10]. Unfortunately, visual diagnosis of subterranean *V. polistiformis* infestations is difficult because many above-ground symptoms of their presence, including fewer and smaller berries, reduced shoot growth, leaf discoloration, and wilting, also can be caused by a multitude of different horticultural and pathological conditions [22] as well as other insects [23].

The timing of emergence of adult *V. polistiformis* can be estimated by monitoring males with pheromone traps [4,8,9]. The timing of larval and pupal stages has been estimated by excavating and inspecting vines for larvae [15], and by counting pupal exuviae under vines [2,3,21]. In Florida, pupal exuviae often are moved from their original positions by fire ants, *Solenopsis invicta* Buren, in which case the vine from which the pupa originated is uncertain; nevertheless, periodic scouting for the presence of new pupal exuviae can be used to estimate the timing of adult emergence [7]. Timing of larval and pupal development in Georgia has been estimated by environmental chamber monitoring of degree days for *V. polistiformis* development stages. Concurrent monitoring of grape berry sugar accumulation using a handheld refractometer has been used to identify host phenological events associated with emergence [24]. Webb et al. [10] reported that in Florida, however, larval development may be correlated more directly with grape root growth, soil temperature, and moisture. Larvae can develop into adults within 1 year [25] in Florida, but development periods can extend up to three years in the northern parts of the species range [3].

Sanders et al. [26] successfully detected the presence of root-feeding *V. polistiformis* larvae in Florida vineyards using methods developed by Brandhorst-Hubbard et al. [27], Zhang et al. [28], and Mankin et al. [29,30,31] for acoustic detection of vibrations produced by insects hidden in soil, trees, and stored products. More recently, acoustic methods have been used to monitor mortality and reduction of activity of larvae exposed to entomopathogenic fungi in situ in palm tree trunks [32,33] and to monitor reductions in activity of stored product insects exposed to hermetic storage treatments [34,35,36]. The distinctive characteristic separating insect larval vibrations from those produced by most other sources is that insect movement and feeding produces bursts of short, broad-band vibrational impulses interspersed with quiet intervals, and the frequency spectra of the vibrations relate consistently to the mechanical and structural characteristics of the insect movement and the substrate [31]. Subterranean larval vibrations are produced by rapid release of energy, e.g., when larval mandibles quickly break or bend plant fibers, or when integument scrapes against the walls of a tunnel that a *V. polistiformis* larva is digging in a grape root. The release of vibrational energy within or to plant structures or soil substrates produces waves [37,38] that spread and are transferred, with damping [39], into other substrates wherever they are making contact. Microphones and mammalian ears can detect longitudinal, compression-rarefaction vibration waves transmitted through air. Because plants and soil resist shear deformation [39], they can transmit longitudinal waves as well as transverse, torsional, bending, and/or surface waves, depending on the nature of the movement and the structure of the substrate, all of which have different transmission speeds and damping factors that can affect the distances over which vibrational bursts can be detected [37,38,39].

Until now, acoustic methods have not been applied to assess seasonal or diel variation of movement and feeding activities of subterranean insects in vineyards. However, temperature is a well-known abiotic factor affecting larval activity of *V. polistiformis* and other insects [10,13,31]. It is known also that grape root growth [40] and amino acid levels in grape roots vary seasonally [41]. Grape root growth and starch levels also vary in circadian rhythm [42]. It would thus not be a surprise to find that *V. polistiformis* larval feeding activity displayed seasonal and circadian variation as well.

For this study, pheromone and acoustic monitoring combined with excavation of soil or inspection of sectors of root systems in the soil near the trunks of vines were conducted in multiple areas of a vineyard at the Florida A&M University (FAMU) Center for Viticulture and Small Fruit Research. Presence of *V. polistiformis* in the vineyard has been reported previously [19,20,21]. The primary objective was to augment visual diagnoses of potential infestation by acoustic identification of vineyard areas containing bursts of vibrations with spectral and temporal patterns previously identified to have been produced by *V. polistiformis* larvae. A second goal was to determine if *V. polistiformis* larval activity varied significantly with abiotic factors such as temperature, season, or time of day, which could affect acoustic detectability. Improved knowledge of the spatial distribution of *V. polistiformis* infestations within a vineyard could assist in the targeting and timing of biological and chemical control treatments.

## 2. Materials and Methods

### 2.1. Vineyard Layout and Grape Varieties

The 18.2 ha vineyard at the FAMU Center for Viticulture and Small Fruit Research has row lengths of 68.4 m, and row spacings of 3 m, with a density of 716 plants/ha [19]. Of 31 different *Vitis rotundifolia* Michaux vines from which acoustic recordings were collected in the study, 22 were Stover variety from the General Management Practices block. Other varieties included Fry, Loomis, Pam, Sterling, Southland, and French–American hybrids. Weed control and preventive sprays of Carbaryl 4 L are performed in spring to reduce the incidence of insect pests. The preventive sprays do not significantly affect subterranean *V. polistiformis* larvae, and adults do not emerge before summer.

### 2.2. Procedures and Timing of Pheromone and Temperature Monitoring, Acoustic Recording, and Vine Inspection

Green Universal Moth Traps (Great Lakes IPM, Vestaburg, MI) baited with female grape root borer (GRB) pheromone [99% (E,Z)-2,13 octadecadienyl acetate, 1% (Z,Z)-3,13 octadecadienyl acetate] (1 mg of pheromone per septa) (Great Lakes IPM, Vestaburg, MI, USA) were placed in four locations at least 50 m apart in the vineyard. A rubber septum with the female GRB pheromone was deposited into each bucket and attached at the top of each trap. Pheromone lures were changed twice per month. A Vaportape (Hercon Environmental, Emigsville, PA, USA) treated with 2,2-dichlorovinyl dimethyl phosphate was affixed to the bottom of each trap to kill the insects. Traps were hung from the trellis wire, 1–1.5 m above the ground, near a vine. Monitoring began in Mid-May of 2014, based on previous studies of emergence timing [10]. Counts of *V. polistiformis* collected in each trap were recorded weekly. Traps were inspected twice weekly until no more *V. polistiformis* were captured.

Acoustic records were collected over a 269-d period, beginning on 9 September 2014 and ending 5 June 2015 using methods described in [30,31]. Concurrent soil and air temperatures were downloaded from the Florida Automated Weather Network station at Quincy, FL, 40 km west of the site. Air and soil temperatures were measured on several occasions to compare with the Florida Network station records and were generally consistent with them. The 269 d were subdivided to separately analyze acoustic activity over three different seasons: fall (0–89 d), winter (90–179 d), and spring (180–269 d). To consider effects of time of day, diel quarters were defined as night (24:00–5:59), morning (6:00–11:59), afternoon (12:00–17:59), and evening 18:00–23:59). As in previous subterranean insect acoustic detection studies [26,27,28,29,30,31], all acoustically monitored root systems were planned for excavation and visual inspection. Because other studies began operation concurrently in the vineyard, however, excavation ultimately was approved for only one site, which contained a visibly unhealthy vine. The vine was excavated after 90 d, at the end of the fall season. One quadrant of the vine at each of five other sites was excavated and the roots examined after the final set of acoustic recordings in spring. As has been noted previously [3], however, the process of removing root systems from the ground provides only an approximate estimate of *V. polistiformis* presence, as portions of the root system typically remain in the soil.

### 2.3. Acoustic Signal Amplification, Sensor Placement, and Inspection of Soil around Sensor

An insect acoustic detection system (Model SP-1L piezoelectric sensor with a 40 kHz resonant crystal, a 1–50 kHz bandwidth, and a 40 dB integral preamp with a 50 ohm output, combined with an AED-2010 amplifier, Acoustic Emission Consulting Inc., Sacramento, CA, USA) and a digital recorder (Model HD-P2, Tascam, Montebello, CA, USA) were set up in the storage bed of an electric cart and transported throughout the vineyard to vines exhibiting symptoms of potential infestation such as wilting, yellowed or dead leaves, and reduced leaf area in comparison with neighboring plants of the same variety. The amplifier was adjustable from 20 to 80 dB, and in most cases, 40–60 dB was used to avoid over-amplifying the signal. This provided sufficient amplification that the type and direction of the wave excitation did not significantly affect the ability to detect insect-produced signals. To serve as a waveguide connecting underground vibrations to the sensor, a 30 cm steel nail was inserted into the ground 10–20 cm from the trunk of a selected vine, in the part of the root system where *V. polistiformis* larvae were most likely to be present [3]. Previous experience indicates that insect larvae producing vibrations in the soil (which is essentially an insulator) can be detected over a distance of approximately 20 cm from the nail [31]. In addition, the grapevine roots serve as a waveguide, carrying signals produced by *V. polistiformis* feeding activity over greater distances in the root system than they are carried through soil. The vine position was noted along with height and width at the first recording. The sensor was attached to the nail head by a magnet. One or more listeners took notes and monitored the signals from invertebrate feeding and movement in the roots during recording periods from 30 s to 3 min. After the recording, the nail was removed and several cubic cm of soil surrounding the nail were inspected to identify arthropods present. After dark, flashlights were used to provide visual assistance to collect arthropods for later identification. In addition, notes were made of above- and below-ground invertebrates observed near recording sites, including calls by crickets and katydids which generated distinctive airborne sounds easily distinguishable from the brief bursts of vibration impulses of *V. polistiformis* and other subterranean invertebrates by their spectra or temporal patterns [31,43,44]. At some sites only a single recording was obtained but, to compare burst rates over time, multiple recordings were obtained from sites where bursts had been detected during the initial test.

The digitization rate was 44.1 kHz with a bit size of 16. The analog signals were band-pass filtered between 1 and 22.05 kHz before digitization to reduce low-frequency background noise and avoid aliasing (where frequencies greater than half the sampling rate are incorrectly interpreted as lower frequencies). To further reduce background noise, recording was avoided during times when wind noise was prominent or the drip irrigation system (Turbo T-tape model 40, 5.0 L/m/h, Rivulis Irrigation Inc. San Diego, CA, USA) was operating.

### 2.4. Digital Signal Processing and Automated Classification of Vibration Impulses

The oscillogram, spectrogram, and audio playback of each recording were prescreened using Raven 1.5 software [45]. The prescreening process confirmed many occurrences of groups (trains) of discrete, 3 to 10 ms impulses separated by intervals <125 ms [31]. Trains containing 7 or more impulses, termed bursts, were a focus of analysis because they often had been identified by listeners as bursts of insect vibrational impulses, i.e., “insect sounds” in playbacks of recordings from infested subterranean sites in this and previous studies [29,30]. During the prescreening, listeners identified four periods of insect signals of distinctly different types produced during intervals relatively free of other types of acoustic signal. Two types of frequently occurring signals, S_low_ and S_high_, were of particular interest during prescreening because they had strong spectral and temporal pattern similarities to those previously detected from confirmed infestations of *V. polistiformis* larvae [26]. Others were confirmed with the listener assessments in the field notes as occurring in the presence or absence of *S. invicta*, *Scudderia furcata* Brunner (katydids), or Gryllinae spp. (crickets). The *S. invicta* could be readily identified in the soil collected around the sensor, and the katydids and crickets could be identified by their loud, distinctive calling songs that are detectable to listeners and also are detected easily by their spectra in the recordings. The airborne calls were detectable as vibrations even though the sensor housing had a foam shield to dampen the transfer of airborne signals to the piezoelectric crystal. The identified signals later were analyzed further to construct mean spectra (profiles) for each of the four signal types, designated as S_high_, S_low_, n_fireant_ and n_orth_, as described in the results below. Periods of long-duration, high-amplitude and low-frequency airborne and soil-borne background noise due to wind, dripping water, or vehicular noise were discarded from further analysis, and relatively noise-free periods of the recordings were analyzed with customized software, DAVIS [46], to further identify insect vibrational bursts and distinguish them from other signals.

The DAVIS program compared a 512-point spectrum [46] of each impulse in the recorded interval with mean spectra (profiles) of known insect vibrational impulses (see *Results*). Each train of impulses was labeled according to the spectral profile matched by a plurality of its impulses. The beginning and ending times of impulse trains, their labels, and the number of impulses per train for each recording were noted in a spreadsheet for further analysis.

### 2.5. Estimation of Infestation Likelihood

Insect larvae produce vibration bursts at highly variable rates over their life cycles [31]; consequently, only a weak relationship was expected between the rates of vibration bursts and the counts of *V. polistiformis* recovered at the site of each recording. Also, only a weak relationship was expected between the rates of vibration bursts and the magnitude of reductions in shoot growth and fruit yield of an infested grapevine. To maximize the guidance that analyses of acoustic recordings are able to provide for management actions, we categorized the likelihood of *V. polistiformis* presence by subdividing vibration burst rates into three broad indicator ranges of low, medium, or high infestation likelihood, as described in [29], and applied the indicator means to specify areas of the vineyard most likely to contain infestations in need of focused control treatment, with lower levels of control treatments adequate elsewhere. The indicator ranges used in this study were derived from previous studies where the insects present at the recording sites had been verified by visual inspection [33,44,47,48]. Indicator variables were set to low, medium, and high likelihood for sites where the combined burst rates for the two types of subterranean invertebrate profile were <0.02 s^−1^, between 0.02 and 0.06 s^−1^, and >0.06 s^−1^, respectively. As in [29], the indicator values for low, medium, and high likelihood were set to 0, 1, and 2, respectively.

The use of infestation indicators is of value for management purposes partly because the amount of damage caused by each *V. polistiformis* larva during its growth cycle is not yet well understood. Individual larvae have been observed to cause relatively minor damage to roots away from the trunk but severe damage when they girdle the trunk and major roots [6]; consequently, estimates of larval counts at individual recording sites cannot yet be used confidently to predict reductions in vine health and crop production.

It should be noted also that, although substrate vibrations produced by moderate- to large-sized *V. polistiformis* larvae can be readily distinguished from those of ants and other small subterranean invertebrates by analyses described in the preceding section, vibrations produced by soil invertebrates of similar size to *V. polistiformis* that have been observed in previous studies [26,28,29], including *Phyllophaga* spp., *Cyclocephala* spp., *Anomala* spp., and Tenebrionids, cannot always be distinguished from one another other without visual confirmation. Differences found in the patterns of stridulatory [49] or scraping [44] activity of the different species ultimately may enable discrimination of the vibrations. However, because their signals cannot yet be discriminated, interpretation of the net effects of activity by these other, similarly sized subterranean larvae requires knowledge of their abundances and spatial distributions. At this time, neither their abundances, spatial distributions, nor their potential for crop damage are well understood in northwestern Florida. However, if the acoustic likelihood of infestation is low, neither *V. polistiformis* nor the other previously found arthropods of similar size are likely to be present in significant numbers. For this reason, it may be necessary for the grove manager to assess whether such insects are present in significant numbers in the soil around grape roots systems and whether their rates of vibration bursts would affect the sizes and numbers of areas considered to have medium or high likelihood of *V. polistiformis* infestation. The concern is addressed further in relation to this specific FAMU vineyard study in the Discussion sections below.

### 2.6. Statistical Analyses

The insect vibration burst rate measurements were found to have a non-normal distribution when tested using the Shapiro–Wilk test in the SAS univariate procedure [50]; consequently, separate non-parametric analyses were conducted on effects of soil temperature, recording site, season, and diel quarter on insect vibration burst rate by applying Kruskal–Wallis χ^2^ tests to the Wilcoxon scores. Non-parametric Steel–Dwass post hoc tests [51] were conducted to compare vibration burst rates among seasons and diel quarters, where the χ^2^ tests were found to be statistically significant.

## 3. Results

### 3.1. Pheromone Trap Monitoring and Inspection of Grape Roots and Soil around Sensor Waveguide

**Figure 1:**
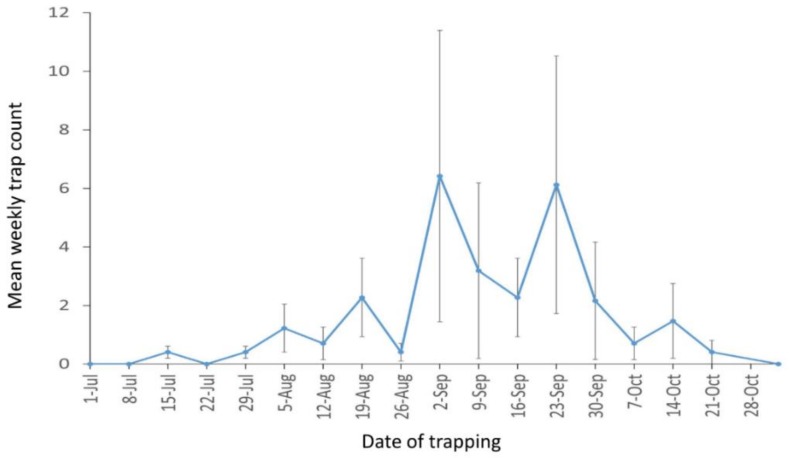
Mean weekly *V. polistiformis* male captures across the 2014 season in Florida A&M University (FAMU) vineyard (standard error of mean indicated by capped bars).

The mean pheromone-trap counts of male *V. polistiformis* collected in different weeks are shown in Figure 1. The first male was captured on 15 July 2014 and none after 5 November 2014. The presence of males in the traps beginning in July suggested that *V. polistiformis* pupal exuviae and larvae would be present in the vineyard by September, which was confirmed at the beginning of the fall acoustic recording measurements. 

No *V. polistiformis* were recovered from any of the five vine quadrant samples examined after the end of the study, but larval burrowing damage was observed in the root system of the vine completely excavated at the end of the fall season. All the excavations contained *S. invicta* workers, including the soil recovered around the sensor waveguide at each recording site. Sound- or vibration-producing organisms found in the soil recovered from the sensor waveguide at the 31 sites included *Scudderia furcata* Brunner (katydids), Gryllinae spp. (crickets), and *Cheiracanthium inclusum* Hentz (spiders). Although previous studies in Florida had recovered *Phyllophaga* spp., *Cyclocephala* spp., *Anomala* spp., and Tenebrionids, none of those species were found in this study.

### 3.2. Mean Spectral Profiles

**Figure 2:**
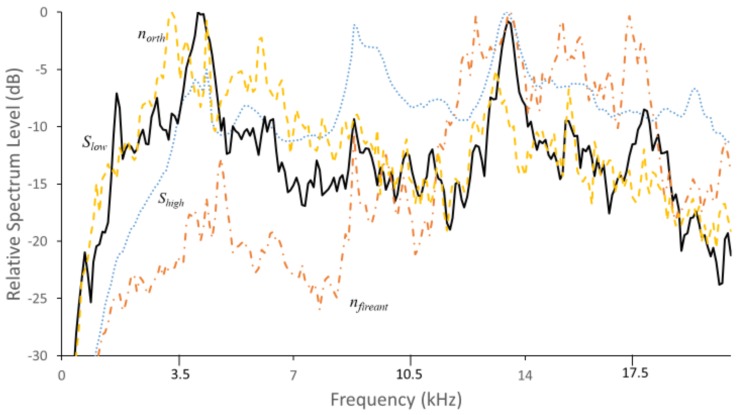
Mean spectra of: S_high_ signals (dotted line) and S_low_ signals (solid line) identified as substrate vibrations of potential subterranean insects in grape root systems; n_fireant_ signals (dot dashed line) identified from fire ants; and n_orth_ signals (dashed line) identified from crickets or katydids.

Mean profiles (average spectra of a series of substrate vibrations or airborne sounds listeners identified to be produced by a single arthropod source) were constructed using the DAVIS insect signal analysis program to enable automated discrimination of insect substrate vibrations and airborne sounds from background noise. Figure 2 shows profiles of the four signal types identified in prescreening to be similar to previously documented *V. polistiformis* signals, S_high_ and S_low_, or signals produced by non-target insects, n_fireant_ or n_orth_. The profile for S_high_ was obtained from a 180 s interval containing 764 impulses with a distinctive spectral pattern and the profile for S_low_ was from a 150 s interval containing 105 impulses of a second distinctive spectral pattern, both of which were judged to be of subterranean larval origin. The profile for n_fireant_ was obtained from a 3 s interval with 8 trains of multiple fire ant vibration impulses, and the profile for n_orth_ was obtained from a 5 s interval with 8 trains of multiple katydid airborne sound impulses. The katydid and cricket signals were similar enough that the n_orth_ profile matched sound impulses produced by both sets of insects. These and the fire ant signals were of lesser interest relative to signals of the targeted *V. polistiformis* and we discarded both types from further analysis.

### 3.3. Insect Vibration Impulse Bursts

Recordings from each of the 31 grapevine root systems in the study were analyzed using the DAVIS insect signal analysis system [46] to identify bursts of S_high_ and S_low_ substrate vibration impulses, and distinguish them from background noise or nontarget signals. The DAVIS software calculated a power spectrum for each vibration impulse with an amplitude above a user-set threshold, and matched it against the spectra of the insect vibration and noise profiles (Figure 2) by calculating the least-squares difference (LSD) between the impulse spectrum and profile signal levels at each spectrum frequency. The impulse was categorized according to the profile type for which the summed LSDs were smallest unless the difference exceeded a user-set threshold, a mean of 10 dB between 1 and 10 kHz, in which case the impulse was discarded as uncategorized noise. Trains of impulses that contained at least 7 of any combination of S_high_ and S_low_ impulses, typical of those produced during larval chewing movements or various digging and scraping activities [31], were classified as insect vibration bursts. 

### 3.4. Mean Vibration Burst Rates at Different Sites across Seasons and Diel Quarters

**Figure 3:**
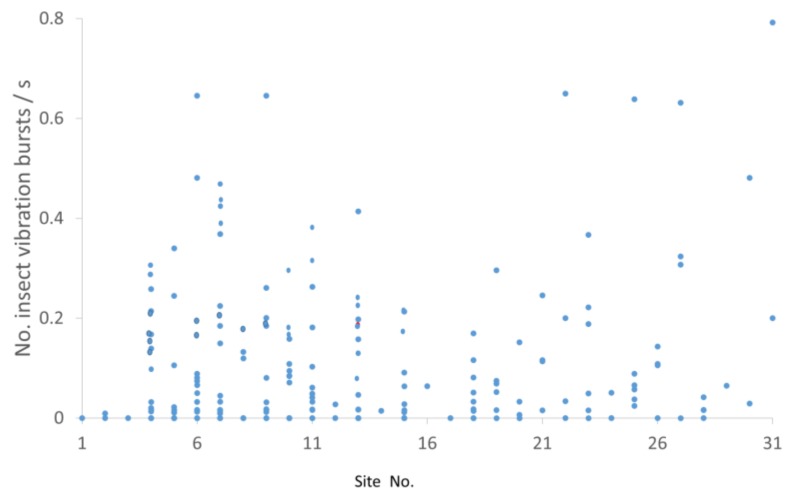
Rates of insect vibration bursts (blue dots) recorded at 31 different vineyard sites during the study.

**Figure 4:**
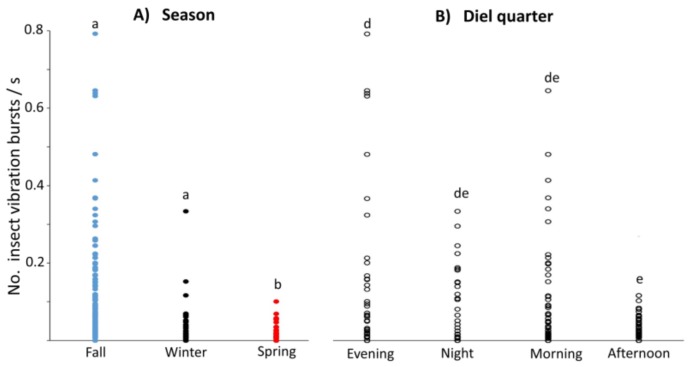
Rates of insect vibration bursts recorded in different (**A**) seasons and (**B**) diel quarters. Rates in different seasons are marked by dots: blue, black, and red for fall, winter, and spring, respectively. Seasons headed by the same letter are not significantly different by the non-parametric Steel–Dwass posthoc test. Rates in different diel quarters are marked by open circles. Diel quarters with headings containing the same letter are not significantly different by the Steel–Dwass test.

Inspection of the spatial, seasonal, and diel quarter patterns of the results suggested that the recording site (Figure 3), season (Figure 4A), and diel quarter (Figure 4B), were significant factors affecting insect vibration burst rates. The Shapiro–Wilk test of normality [48] revealed that the distribution of insect vibration burst rates was non-normal (N = 309, W = 0.549052, *p* < 0.0001); therefore, nonparametric analyses using the Kruskal–Wallis χ^2^ test were conducted. The effects of soil temperature were not found to be statistically significant and will not be further discussed. The effects of site, season, and diel quarter were found to be statistically significant, with df = 30, χ^2^ = 48.67, and *p* < 0.017 for site; df = 2, χ^2^ = 26.78 and *p* < 0.001 for season; and df = 3, χ^2^ = 9.56, *p* = 0.023 for diel quarter. Consequently, nonparametric posthoc comparisons of winter, fall, and spring burst rates were conducted using the Steel–Dwass test for all seasonal pairs [51]. Of these, the comparison between fall and spring (Z = 4.94, *p* < 0.001) and between winter and spring (Z = 2.63, *p* = 0.023) were statistically significant. Non-parametric post hoc comparisons of evening, night, morning, and afternoon burst rates were conducted also using the Steel–Dwass test, and the only statistically significant comparison was between evening and afternoon (Z = 2.97, *p* = 0.016).

### 3.5. Spatial and Temporal Assessment of the Likelihood of Infestation

**Figure 5:**
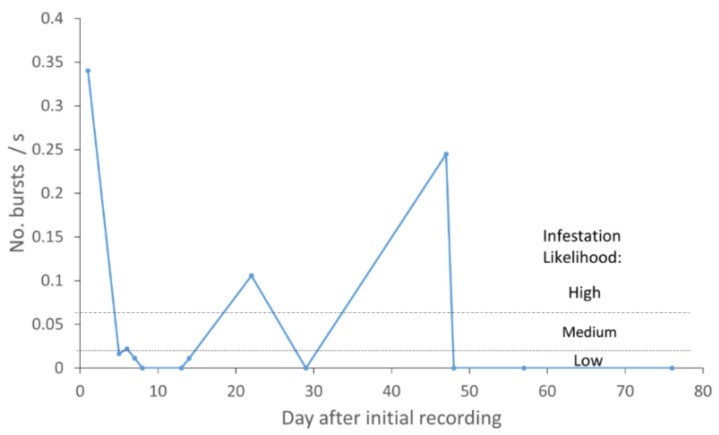
Insect vibration burst rates (marked by dots on solid line) at recording site where root system revealed *V. polistiformis* larval damage. Rates below 0.02 bursts s^−1^ (indicated by the dashed line) are rated at low infestation likelihood, rates above or at 0.06 bursts s^−1^ (indicated by the dash-dotted line) are rated high, and rates between 0.02 and 0.06 bursts s^−1^ are rated medium.

The various rates of insect vibration bursts detected at the 31 recording sites in the vineyard are plotted in Figure 3 to display the typical variability of vibration burst rates at individual sites monitored multiple times. The high variability observed in Figure 3 complicates analysis of the acoustic information obtained from individual sites when the goal is to estimate the likelihood of infestation in different areas of the vineyard. An example of the interpretation of burst activity in terms of infestation likelihood indicators is provided in Figure 5, where insect vibration burst rates were plotted from recordings obtained on different days at site 5 in Figure 3, where larval damage was confirmed by excavation. The pattern of burst rates over time suggests that a larva may have been feeding in the root system but moved elsewhere or died by day 48 after the initial recording, after which no bursts were detected. The periods of low infestation likelihood observed near days 10 and 29 could have occurred when a larva moved away and then back to the root, or they could have occurred when the larva molted, as reported for other species in [33,48]. A total of six instars has been reported in *V. polistiformis* [7]. In four other recordings, this site was rated at medium (1) or high (2) infestation likelihood up to day 48.

**Figure 6:**
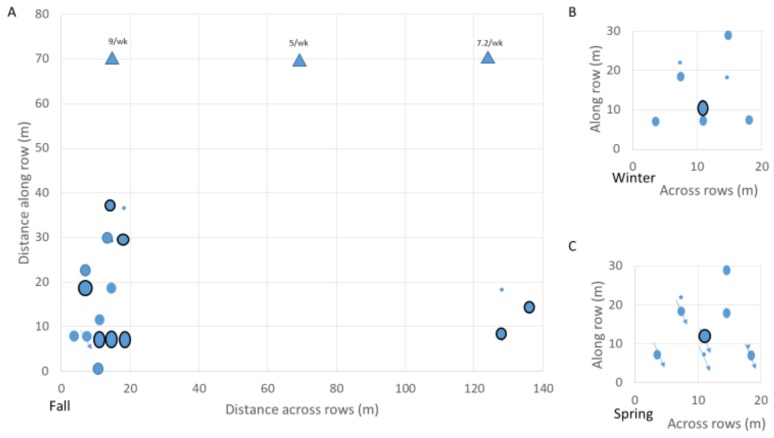
Infestation likelihood indicator values during (**A**) fall, (**B**) winter, and (**C**) spring seasons in vineyard sites of greatest activity: small ovals indicate mean between 0 and 0.5 (low infestation likelihood); larger ovals, 0.5 to 1.5 (medium infestation likelihood); and ovals with black outline, mean >1.5 (high infestation likelihood); arrows mark sites where soil was excavated to identify grape root borer (GRB) larvae or larval damage to roots; and triangles denote location of three pheromone traps near the recording sites. Mean weekly trap counts are listed above trap location. Means at 17 sites are shown in (**A**), 8 in (**B**), and 8 in (**C**).

Over the 269 d experiment, 121 recordings (39% of 309 in total) indicated low infestation likelihood. Mean values of the indicator variables for infestation likelihood averaged within individual sites are shown by season in Figure 6 for the most active parts of the vineyard. The highest mean values for infestation likelihood during fall were recorded in the first 30 m of five rows between 10–20 m from the edge of the vineyard section, and activity remained in 8 of those vines during winter and spring. Because the excavated vine with root damage was present in this area, both the acoustic and excavation methods were in agreement about the presence of *V. polistiformis* infestation in this area.

## 4. Discussion

Acoustic detection of subterranean arthropods in vineyards remains primarily a research tool rather than a widely used measurement system for assessing *V. polistiformis* damage in grapevine root systems, and the results of this study illustrate several uncertainties and ambiguities that often are encountered in research on management of subterranean insect pests. Until now, the focus of *V. polistiformis* acoustic detection research has been to develop methods of confirming its presence in areas where visual or other evidence had identified potential vineyard infestation. In this study for example, presence of exuviae under the grape vines when the study began in September and captures of males in pheromone traps at the edge of the vineyard beginning earlier in July provided initial evidence that *V. polistiformis* larvae might be present. The temporal pattern and mean counts of males per trap per week in Figure 1 are consistent with those observed at FAMU between 2008 and 2012 by [19] and those observed near FAMU in 2012–2013 by [4], suggesting the frequent reintroduction of *V. polistiformis* into the vineyard. Two potential areas of infestation were found by visual identification of vines with wilting or discolored leaves [3]. Recordings in the soil under vines in one of the two areas detected vibration bursts of appropriate spectral and temporal patterns at rates that indicated a medium or high likelihood of *V. polistiformis* infestation. Recordings in this area continued through winter and spring to consider whether effects of temperature, season and time of day affected acoustic detectability. By the end of the study, six sites continued to be acoustically rated at medium to high likelihood of infestation (Figure 6C).

Execution of the original experimental plan was altered when permission was granted to fully excavate only one vine in the area of greatest activity to verify the acoustic identification by complete excavation and visual inspection. The presence of *V. polistiformis* damage in this vine was confirmed, but this result did not necessarily provide strong confirmation also for the estimations of medium or high infestation from recordings beneath other vines nearby. Ambiguity remained about the presence of grass-feeding *Phyllophaga*, *Cyclocephala*, and *Anomala* spp. observed previously in Florida, although no larvae of any of these species were recovered from excavations around the sensor waveguide after any of the recordings. However, it is not surprising that these insects were absent in unsheltered areas of a vineyard where *S. invicta* were abundant. Fire ants have been reported to prey frequently on Coleopteran larvae [52].

For such reasons, it is necessary to characterize this report as partly a discussion of difficulties in how to derive useful information from a field study where many of the variables cannot always be experimentally controlled. Many such unknowns encountered in attempting research on *V. polistiformis* larvae and other subterranean insect pests, e.g., [53], continue to be reported in the literature. Such uncertainties highlight the need for additional experimental methods to relate the behavior of subterranean insect pests more precisely with factors affecting grapevine health and productivity.

One path forward could be to monitor productivity of grapevines in an environmentally controlled screened cage by several methods before and after exposure to *V. polistiformis* neonates while simultaneously the soil around the root system of each vine is acoustically monitored continuously at multiple locations over the insect life cycle. Subsequently, the root system could be examined for quantification of insect feeding damage. Such procedures also would help quantify the fraction of time the larva spends feeding, moving, resting, and molting. The time budget of *V. polistiformis* larval activity remains largely unknown. Better understanding of the larval activity budget and foraging patterns could clarify understanding of the factors that affect its growth and damage to the grape root system. Similar studies could be conducted with other subterranean arthropods of similar size to *V. polistiformis* in vineyards where they may be present in abundance.

A second research path could be to identify and implement alternatives to the excavation of grapevine root system to assess the presence of *V. polistiformis*. One potential solution is to construct playback devices that emit *V. polistiformis* movement and feeding sounds and can be set up near recording sites to confirm that the acoustic sensors successfully detect playback signals from various distances. This would enable separate validation of sensor function in a control test without insects. Similarly, a bucket of sterilized soil may be a preferred negative control for insects, because it is not likely that a randomly selected recording site in a field can be guaranteed to be free of acoustically active subterranean arthropods. Also, experience suggests that frequent exposure to insect larval vibrations and interfering signals in the vineyard can assist users of acoustic sensors in learning to discriminate signals produced by target insects from above-ground insect, bird, or human noise, and vehicle or irrigation equipment noise. A combination of such methods ultimately may serve as an alternative to the current usage of direct inspection of grapevines for confirmation of *V. polistiformis* infestation.

Keeping in mind the uncertainties above, the results obtained from this study suggest, nevertheless, that future *V. polistiformis* management efforts may benefit from acoustic studies that focus on identification of aggregations of subterranean arthropod activity. Index values of medium or high infestation likelihood were aggregated within small areas of the FAMU vineyard (Figure 6) and future studies may similarly identify areas with consistently low infestation likelihood that could receive no or low levels of treatment. Indeed, a nonrandom distribution of acoustically monitored vineyard sites with high infestation likelihood was not unexpected in this study. Previous investigations frequently have found distributions of multiple species of insects to be spatially aggregated rather than random or uniform [27,29,54,55,56]. 

Even without labor-intensive, costly root inspection of each recording site, the study results suggest opportunities for further application of acoustic methods to develop useful information about interactions between *V. polistiformis* larvae and grape vine root systems that have been difficult to investigate previously. For example, variation observed in mean insect vibration burst rates during different seasons and diel quarters might have been expected to result from soil temperature changes, given that insect activity typically increases as the temperature increases above 10 °C [31,57,58,59]. The observed pattern in this study suggests, however, that other factors contributed to the observed variability of insect activity. Vibration burst rates were significantly lower in afternoon than evening, although the mean temperature was warmer in the afternoon. Effects of the grapevine circadian rhythm may have played a role in the difference between evening and afternoon burst rates. Although a subterranean insect like *V. polistiformis* does not sense photoperiod directly, grapevines [60] and other plants [61] have circadian clocks that modulate daily phenological events, including photosynthesis, and opening and closing of stomatal pores. It may not be coincidental that the evening period of greatest acoustic activity corresponds to the daily period of fastest grape root growth [42]. Plant photosynthetic cycles previously have been observed to affect growth and dieback of plant symbionts [62]. Models of optimal foraging [63,64] suggest that herbivorous insect larvae would maximize fitness by feeding on the highest quality food, i.e., *V. polistiformis* larvae might focus their feeding activity on new growth during the evening rather than feeding constantly, independent of nutrient quality, over the daily cycle.

Similarly, the mean insect vibration burst rate was found to be significantly lower in spring than fall and winter although temperatures were higher in spring. These reduced levels of vibration burst rates are consistent with spring and early summer changes in *V. polistiformis* development [24] as larvae pupate over a 30–45 d period and adults emerge [4,7]. Reductions of insect activity during metamorphosis and pupation have been noted in previous studies [33,48,57]. The timing of pheromone trap counts in Figure 1, suggests that these adults began pupating from late May to August. Such patterns of seasonal and time of day acoustic activity patterns would not be expected in other subterranean arthropods that do not feed on grapevine roots or have different life cycles than *V. polistiformis*. 

Overall, the study results suggest that a preferred time to acoustically screen individual root systems for presence of *V. polistiformis* is fall evenings. Further studies are recommended to address the magnitude of seasonal and diel period factors on *V. polistiformis* behavior more precisely. North of Florida, seasonal patterns of *V. polistiformis* activity likely would vary according to differences in temperature, rainfall, and the phenological characteristics of different grape varieties. Development of regional knowledge of such patterns could help guide management decisions to mitigate damage from this important vineyard pest in the southeastern US.

## 5. Conclusions

Acoustic signal analyses of subterranean recordings collected near the trunks of grape vines at different sites in a FAMU vineyard indicated that vibration bursts potentially originating from *V. polistiformis* larvae were spatially aggregated in the vineyard, and the burst rates varied over season and diel period. Activity was significantly greater in fall and winter than in spring, in correlation with the season of greatest grape root growth. Activity was significantly greater in evening than afternoon, in correlation with the diel period of greatest root growth. The results suggest that fall evenings are the best time to acoustically survey Florida vineyards for *V. polistiformis*. When *V. polistiformis* is spatially aggregated, there is potential to employ acoustic surveys to reduce the coverage of management treatments. Such knowledge may be of value for guidance of *V. polistiformis* management decisions if the results of the study are confirmed under multiple different environmental conditions. It should be noted, however, that expenses associated with robustly replicated acoustic surveys reduce their feasibility of general use to predict individual or groups of vines that need control treatments and those that do not. In addition, the use of acoustic surveys to understand long-term ecological interactions between *V. polistiformis* larvae and grape vine root systems has barely begun, and the findings reported here need to be further expanded and replicated.
